# *STAC3* disorder: a common cause of congenital hypotonia in Southern African patients

**DOI:** 10.1038/s41431-024-01644-5

**Published:** 2024-06-01

**Authors:** Fahmida Essop, Bronwyn Dillon, Felicity Mhlongo, Louisa Bhengu, Thirona Naicker, Lindsay Lambie, Liani Smit, Karen Fieggen, Anneline Lochan, Jessica Dawson, Phelelani Mpangase, Marc Hauptfleisch, Gail Scher, Odirile Tabane, Marelize Immelman, Michael Urban, Amanda Krause

**Affiliations:** 1 Division of Human Genetics, National Health Laboratory Service and School of Pathology, The University of the Witwatersrand, Johannesburg, South Africa; 2 Genetics, Department of Paediatrics, Inkosi Albert Luthuli Central Hospital and University of KwaZulu-Natal, Durban, South Africa; 3 Genetics Department, Ampath National Reference Laboratory, Centurion, South Africa; 4 Department of Obstetrics and Gynaecology, Faculty of Medicine and Health Sciences, Stellenbosch University, Stellenbosch, South Africa; 5 Division of Human Genetics and Department of Medicine, University of Cape Town, Cape Town, South Africa; 6 Department of Medical Genetics, University of British Columbia, Vancouver, BC Canada; 7 Sydney Brenner Institute for Molecular Bioscience, University of the Witwatersrand, Johannesburg, South Africa; 8 Department of Paediatrics, Faculty of Health Sciences, School of Clinical Medicine, Chris Hani Baragwanath Academic Hospital, The University of the Witwatersrand, Johannesburg, South Africa; 9 National Health Laboratory Service Human Genetics Laboratory, Groote Schuur Hospital, Cape Town, South Africa

**Keywords:** Disease genetics, Pathology, Mutation

## Abstract

*STAC3* disorder, or Native American myopathy, is characterised by congenital myopathy, hypotonia, musculoskeletal and palatal anomalies, and susceptibility to malignant hyperthermia. A *STAC3* c.851 G > C (p.Trp284Ser) pathogenic variant, common in the Lumbee Native American tribe, has been identified in other populations worldwide, including patients of African ancestry. We report on the frequency of *STAC3* c.851 G > C in a cohort of 127 patients presenting with congenital hypotonia that tested negative for spinal muscular atrophy and/or Prader-Willi syndrome. We present a clinical retrospective, descriptive review on 31 Southern African patients homozygous for *STAC3* c.851 G > C. The frequencies of various phenotypic characteristics were calculated. In total, 25/127 (20%) laboratory-based samples were homozygous for *STAC3* c.851 G > C. A carrier rate of 1/56 and a predicted birth rate of 1/12 500 was estimated from a healthy cohort. A common haplotype spanning *STAC3* was identified in four patients. Of the clinical group, 93% had a palatal abnormality, 52% a spinal anomaly, 59% had talipes equinovarus deformity/deformities, 38% had arthrogryposis multiplex congenita, and 22% had a history suggestive of malignant hyperthermia. The novel finding that *STAC3* disorder is a common African myopathy has important clinical implications for the diagnosis, treatment and genetic counselling of individuals, with neonatal and/or childhood hypotonia with or without arthrogryposis multiplex congenita, and their families. The spread of this variant worldwide and the allele frequency higher in the African/African-American ancestry than the Admixed Americans, strongly indicates that the *STAC3* c.851 G > C variant has an African origin which may be due to an ancient mutation with migration and population bottlenecks.

## Introduction

*STAC3* disorder (*STAC3* [MIM #255995]), previously known as Native American myopathy (NAM), is a congenital myopathy first described by Bailey and Bloch in 1987 in a three-month-old infant of American Indian Lumbee tribe descent [1]. In 1988, Stewart et al. [2] described an additional six children of Lumbee descent with features of a myopathy, cleft palate, skeletal anomalies, and susceptibility to malignant hyperthermia (MH) and suggested a likely autosomal recessive inheritance pattern for this condition. Approximately twenty years later, in 2008, Stamm et al. [3] identified a common homozygous region on chromosome 12q13.13-q14.1 through homozygosity mapping of five Lumbee families. This was followed by the identification of homozygous biallelic pathogenic variants in the *STAC3* (SH3 and cysteine-rich domain protein 3) gene and the c.851 G > C (p.Trp284Ser) missense variant (rs140291094) as a cause of NAM [4]. The STAC3 protein is critical for skeletal muscle excitation-contraction (EC) coupling, vital for the release of Ca^2+^ that leads to muscle contraction and recently, in 2022, STAC3 was shown to modulate the current kinetics of the skeletal muscle calcium channel CaV1.1 [4–6].

**Fig. 1:**
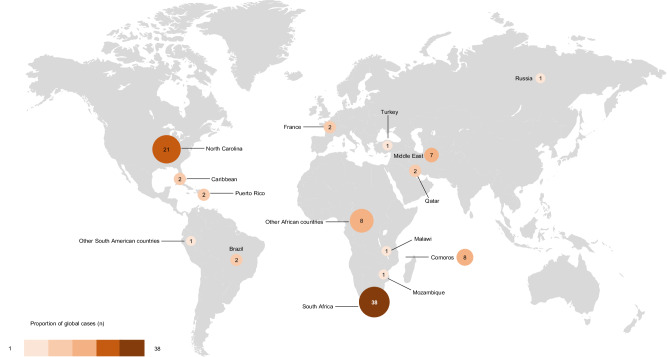
The map illustrates the geographic distribution of all reported *STAC3* disorder cases resulting from the *STAC3* c.851 G > C (p.Trp284Ser) pathogenic variant published worldwide to date. The size of each circle corresponds to the number of reported cases within the respective country, while the colour gradient indicates the proportion of global cases attributed to each country. The majority of cases (*n* = 38) were reported in South Africa, including 36 cases from this study. This includes 25 cases from the laboratory cohort, 18 of which are described in the clinical cohort, and an additional 11 cases from the clinical cohort.

As the Lumbee tribe has a high rate of consanguinity and is a culturally isolated group, it was proposed that the high frequency of *STAC3* disorder was due to a local founder effect [3]. The c.851 G > C variant, together with other *STAC3* variants have since been described in other diverse population groups worldwide, as illustrated in Fig. 1. This includes a consanguineous Qatari family, Puerto Rican family and patients of African, Afro-Caribbean, Comorian, Middle Eastern, Russian and South American origin [7–14], suggesting the *STAC3* c.851 G > C variant is more widely distributed geographically.

Here we present the largest cohort of patients identified with *STAC3* disorder, all predominantly of African ancestry and originating from Southern Africa. All were shown to be homozygous for the NAM pathogenic variant c.851 G > C. In addition, we investigated the clinical phenotype of these African patients, to determine the typical phenotypic findings, and to determine if the features and their frequencies are consistent with those reported in other *STAC3* cohorts worldwide.

## Materials and methods

Two overlapping cohorts were studied and are described below. Firstly, a laboratory-based cohort was used to ascertain the contribution of *STAC3* disorder to the diagnosis of hypotonic infants and children. Once *STAC3* disorder was determined to be a common diagnosis, a patient cohort was identified to study the clinical phenotype.

### Laboratory cohort

After two Southern African patients were identified on a new Next generation sequencing (NGS) neuromuscular panel to be homozygous for *STAC3* c.851 G > C, studies were initiated to determine whether this variant could be retrospectively identified in patients previously receiving genetic testing for infantile and childhood hypotonia. Prior testing was limited to spinal muscular atrophy (SMA) and Prader-Willi syndrome (PWS). A cohort of unrelated patients of African ancestry who initially tested negative for SMA (*n* = 114) or PWS (*n* = 13) were screened for *STAC3* c.851 G > C in the Human Genetics Laboratory, National Health Laboratory Service, Johannesburg, South Africa. The patients were mostly ages 0–5 years and were referred to the Division between 2012 and 2022 from paediatric neurology clinics in South African State hospitals, predominantly in Southern Gauteng and KwaZulu-Natal. The *STAC3* c.851 G > C variant located in exon 10 (NM_145064.3) was detected using targeted Sanger sequencing.

### Clinical cohort

The clinical cohort consisted of 31 homozygous *STAC3* c.851 G > C variant positive patients, ascertained from genetic clinics across South Africa, both in the public health sector, and recruited through a private pathology laboratory. A retrospective, descriptive review was performed on the patients. Comprehensive clinical notes were not available for all patients and the number of patients with available data varied across the characteristic categories. Thus positive features were possibly more likely to be reported than their absence. Data were entered into a REDCap database [15, 16]. The frequency of various phenotypic characteristics was calculated and reported.

### Haplotype analysis

Haplotype analysis was not performed on all samples, due to resource limitations. However, five patients had chromosomal microarray analysis for diagnostic purposes that included probes for single nucleotide polymorphisms (SNPs), using Cytoscan Optima Suite (Thermo Fisher Scientific, Waltham, US). The array includes 148,450 SNP markers uniformly spaced over the genome, which were assessed for evidence of regions of homozygosity encompassing the *STAC3* gene, and for the presence of a common haplotype. The 25 laboratory-based samples homozygous for *STAC3* c.851 G > C were inspected for four SNPs, rs138921555, rs367701062, rs142117531 and rs115276341 included in the Sanger sequencing amplicon [17].

### Allele frequency and carrier rate

The frequency of *STAC3* c.851 G > C was estimated in the Southern African population using available whole exome sequencing (WES) data from 278 healthy unrelated individuals in the Deciphering Developmental Disorders in Africa (DDD-Africa) study [18]. The individuals are of similar ancestry to the *STAC3* disorder cohort. All 278 VCF files were combined into a single VCF file using the “bcftools merge” to obtain the minor allele frequency (MAF) for c.851 G > C [19]. The VCF was passed to PLINK to obtain the heterozygote frequency [20].

The allele frequencies were compared between the DDD-Africa cohort and the African/African American allele frequency reported in gnomAD v4.0.0 variant ID 12-57244322-C-G (GRCh38) [21] using a Fisher’s exact test.

## Results

### Molecular analysis

Of the 127 patients tested, 25 were homozygous for *STAC3* c.851 G > C, a diagnostic yield of 20% (25/127). Of these, 22 of 114 were previously tested for SMA which was the primary cohort screened, and three of 13 patients underwent testing for PWS.

### Allele frequency and carrier rate

Of the 278 samples screened in the DDD-Africa cohort for *STAC3* c.851 G > C, we identified five heterozygotes (carrier frequency 5/278 = 1.8%, 95% confidence interval 0.77–4.14%; allele frequency 5/556 = 0.899%, 95% CI 0.38% to 2.09%). The variant occurs at a very low frequency in gnomAD v4.0.0, with the highest allele frequency being in Africans/African Americans at 0.001 (75/75066). It is also reported in Latino/Admixed Americans at 0.0001333, and Europeans at 0.000008. Fisher’s exact test comparison revealed a statistically significant allele frequency difference between the DDD-Africa cohort and the African/African American allele frequency reported in gnomAD v4.0.0 (*p*-value = 3 ×10^−^^4^).

### Haplotype analysis

Haplotype analysis of five patients tested on a diagnostic SNP array, all homozygous for *STAC3* c.851 G > C, showed overlapping regions of homozygosity (ROH) that encompassed the *STAC3* gene. Four share a ROH spanning 1.8 Mb, with the fifth sharing 442 kb including the area of the *STAC3* locus. The details are provided in the supplementary table (Table 4). As expected, all four SNPs in the *STAC3* exon 10 region amplified on Sanger sequencing were homozygous and all shared the same alleles.

### Clinical analysis

#### Demographic information

Of the 31 unrelated patients; there were 19 (61%) males and 12 (39%) females. All were from Southern Africa with African ancestry, of various Southern African ethnolinguistic groups including Sepedi, Sotho, Tswana, Tsonga, Xhosa, Zulu, Chichewa, and Shona. The median age at which clinical data were obtained was 40 months (range 2 days–17 years). At the time of data collection 18/31 (58%) patients were alive, 3/31 (10%) had died and the status of the remaining 10/31 (32%) patients was unknown. The ages of death were one week, 19 months and two years. Two of the deaths were due to respiratory complications, and not documented for the third patient. No parental consanguinity was reported in 25 patients for whom data were available.

#### Perinatal information

**Fig. 2:**
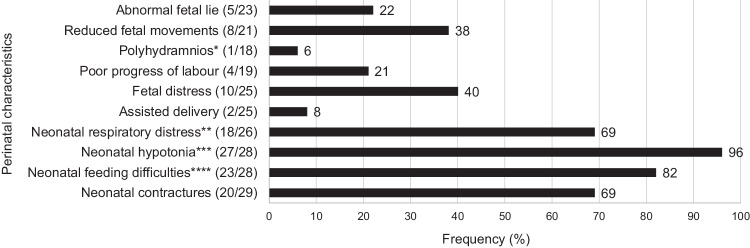
Frequency of major perinatal characteristics of Southern African patients of African ancestry with *STAC3* disorder due to apparent homozygosity for the S*TAC3* pathogenic variant (c.851 G > C, p.Trp284Ser). **Notes**: *Only one patient had documented polyhydramnios, although it is unknown how many patients had prenatal ultrasound examination. **Ranging in severity (subjectively graded as mild (8/18 (44%)), moderate 4/18 (22%), severe (1/18 (6%), or unknown 5/18 (28%))). ***Ranging in severity (subjectively graded as mild (6/27 (22%)), moderate 6/27 (22%), severe 10/27 (37%), or unknown 5/27 (19%)). ****Feeding difficulties included poor suck and/or difficulty swallowing.

Major perinatal characteristics are summarised in Fig. 2, with details in the supplementary table (Table 3). Twenty-five (25/29 (86%)) patients were born at term gestation and 4/29 (14%) prematurely. A high frequency of patients (18/26 (69%)) experienced respiratory distress at birth, ranging in severity (subjectively graded as mild (8/18 (44%)), moderate (4/18 (22%)), or severe (1/18 (6%))). A weak cry at birth (16/17 (94%)) and neonatal hypotonia (27/28 (96%)) were almost universally present in the patients. Neonatal hypotonia ranged in severity from mild (6/27 (22%)) to severe (10/27 (37%)), based on subjective clinical judgement. A high frequency (20/29 (69%)) of neonatal contractures was noted; 17/29 (59%) were noted to have unilateral/bilateral talipes equinovarus (TEV) deformities, 12/30 (40%) had camptodactyly, and 11/29 (38%) had two or more joint contractures.

### Clinical features

#### Growth

Almost half of the patients were born small for gestational age (SGA) (11/25 (44%)). The remaining patients were born appropriate for gestational age (14/25 (56%)). At birth, the majority (13/18 (72%)) of patients were recorded as having a length within the normal range (between the 3rd and 97th centiles), and 3/18 (17%) patients had short stature defined by length below the 3rd centile; two patients had length between −2.00 and −3.00 standard deviation (SD) scores and one patient had a length greater than 3.00 SD below the mean. At birth most patients (18/19 (95%)) had head circumference measurements within the normal ranges (3rd to 97th centile), and one patient (1/19 (5%)) had macrocephaly (greater than three SD scores above the mean) of unknown/undocumented cause. Gestational ages were known for all patients and growth measurements were corrected for prematurity where necessary.

At the time of their last clinical visits (at a median age of 926 days, interquartile range 1601 days) the majority (16/23 (70%)) of patients were underweight-for-age (weight below −2 SD), and 10/23 (43%) were severely underweight for age (weight below −3 SD). The remainder (7/23 (30%)) had normal weight for age. More patients (12/22 (55%)) were of normal stature; however, close to half of the patients (10/22 (45%)) were noted to be of short stature both below the 3rd centile and below −2 SD. Failure to thrive (FTT) was noted in 17/26 (65%) patients. Most patients (19/24 (79%)) had normal head circumference measurements at the time of their last clinical visit; the remaining 5/24 (21%), had head circumference measurements less than the 3rd centile and below −2 SD, which was of postnatal onset in two patients, and of unknown onset in three patients.

#### Craniofacial and systemic features

**Fig. 3:**
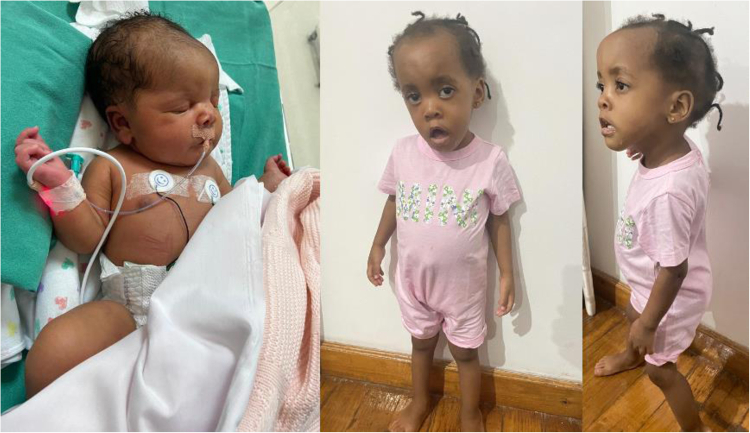
A female patient of Southern African ancestry with confirmed *STAC3* disorder in neonatal period (left) and at approximately two years old (middle and right). Left: note the hypotonic posture and orogastric feeding tube in situ. Middle and right: note the long, expressionless face, tented upper lip, and open mouth posture.

**Fig. 4:**
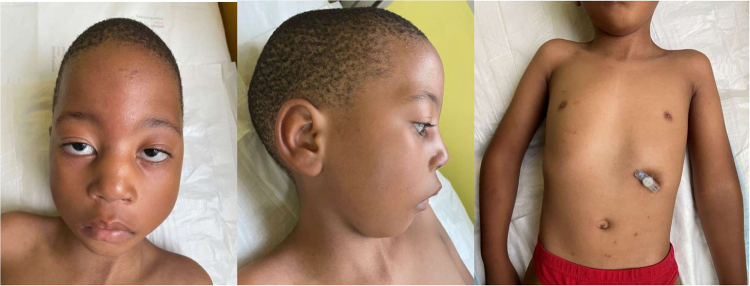
A male patient of Southern African ancestry with confirmed *STAC3* disorder at 4 years of age. Left and middle: note the long, expressionless face, downturned corners of the mouth, and retrognathia. Right: note the percutaneous gastrostomy feeding tube in situ.

Dysmorphic features were recorded in 30/30 (100%) and are detailed in Table 1 and demonstrated in Figs. 3 and 4. Palatal abnormalities were noted at a high frequency, in 26/28 (93%); 11/28 (39%) had a cleft palate and 16/28 (57%) had a high-arched palate. Systemic features are detailed in Table 1. Respiratory difficulties that extended beyond, or occurred after the neonatal period, were noted in almost half of the patients (7/16 (44%)). Of these patients 1/7 (14%) required continuous positive airway pressure ventilation, 2/7 (29%) required nasal prong oxygen (of unknown concentration and flow) and the intervention required was unknown in 4/7 (57%) patients. Structural cardiovascular anomalies (patent ductus arteriosus and atrial septal defect) were noted in 2/18 (11%) patients, both of whom were born at term gestation. Cardiovascular abnormalities were recorded as not present in 16/18 (89%) patients, although only six of these were based on formal echocardiographic assessment. There was no evidence of neurodegeneration in the present cohort.

**Table 1:** Craniofacial features, systemic abnormalities and disease characteristics noted in Southern African patients of African ancestry with *STAC3* disorder due to apparent homozygosity for the S*TAC3* pathogenic variant (c.851 G > C, p.Trp284Ser), compared where possible to cohorts from Stamm et al. (3), Zaharieva et al. (12) and Gromand et al. (7).

Clinical feature			Number (n/N)(%)			
			Present study	Stamm et al. (2008)	Zaharieva et al. (2018)	Gromand et al. (2022)
**Craniofacial features**	**Skull**	Plagiocephaly	5/30 (17)			
	**Ears**	Low-set ears Posteriorly rotated ears	8/30 (27) 6/30 (20)			
	**Eyes**	Abnormality of eye morphology	18/30 (60)			5/7 (71)
		Short palpebral fissures	1/30 (3)			
		Down-slanted palpebral fissures	0/30 (0)			
		Ptosis^a^	16/30 (53)	8/10 (80)	18/18 (100)	4/7 (57)
		Epicanthic folds	7/30 (23)			1/7 (14)
	**Oral**	Downturned corners of the mouth	6/30 (20)	6/7 (86)		2/7 (29)
		Mouth held open	6/30 (20)			3/7 (43)
		Tented upper lip	14/30 (47)			
		Micrognathia	3/30 (10)	7/14 (50)		
		Retrognathia	2/30 (7)			
		Microretrognathia	6/30 (20)			
		Palatal abnormality^b^	26/28 (93)	14/14 (100)	10/18 (56)	
		High-arched palate	16/28 (57)	5/14 (36)		
		Cleft palate	11/28 (39)	9/14 (64)		7/7 (100)
	**Neck**	Short neck	3/30 (10)			
**Systemic feature**	**Chest**	Chest wall abnormality	13/28 (46)			
		Pectus excavatum	1/28 (4)			
		Pectus carinatum	7/28 (25)			
		Narrow chest	2/28 (7)			
		Other^c^	3/28 (11)			
	**Respiratory**	Respiratory difficulty^d^	7/16 (44)			
	**Cardiovascular**	Structural cardiovascular abnormality^e^	2/18 (11)			
	**Abdominal**	Inguinal hernia	1/27 (4)			
	**Genital**	Females				
		Normal external genitalia	11/11 (100)			
		Males				
		Normal genitalia	9/16 (56)			
		Unilateral/bilateral cryptorchidism	7/16 (44)		7/11 (64)	1/5 (20)
	**Skin**	Pigmentary abnormality^f^	3/27 (11)			
	**Spine**	Abnormality present	14/27 (52)		11/18 (61)	
		Scoliosis^g^	8/27 (30)			
		Kyphosis	2/27 (7)			
		Kyphoscoliosis	3/27 (11)			
		Abnormality not specified	1/27 (4)			
	**Limbs**	Camptodactyly	12/30 (40)			4/7 (57)
		Overlapping fingers	1/29 (3)			
		Reduced finger/palmar creases	4/29 (14)			
		Wrist joint contracture/s	0/29 (0)			
		Elbow joint contracture/s	2/29 (7)			
		Shoulder contracture/s	0/29 (0)			
		Hip joint contracture	1/29 (3)			
		Knee contracture	0/29 (0)			
		Talipes equinovarus^h^/history of	17/29 (59)	10/13 (77)	12/18 (67)	5/7 (71)
		Genu recurvatum^h^	2/29 (7)		7/15 (47)	
		Joint hyperlaxity^i^	4/29 (14)			
		Genu valgus/varus	0/29 (0)			
		Pes planus/cavus	0/29 (0)			
		Overlapping toes	4/29 (14)			
		Pterygium	0/29 (0)			
		Arthrogryposis multiplex congenita^j^	11/29 (38)			
	**Neurological/ neuromuscular**	Abnormality present	31/31 (100)			
		Myopathic facies	27/28 (96)	14/14 (100)		7/7 (100)
		Reduced muscle bulk	25/29 (86)			
		Hypotonia	31/31 (100)			
		Reduced power (global)	22/27 (81)			
		Reduced power (proximal>distal)	3/27 (11)			
		Ophthalmoplegia	3/24 (13)		0/18 (0)	
		Tongue fasciculations	3/28 (11)			
		Swallowing difficulties	13/24 (54)			
		Speech impairment^k^	13/17 (76)			
		Areflexia (global)	6/29 (21)			
		Hyporeflexia (global)	18/29 (62)			
		Developmental delay^l^	27/27 (100)			
		Global developmental delay	23/27 (85)			
		Motor delay only	4/27 (15)			
		Intellectual disability^m^	2/6 (33)			
	**Other clinical**	Hearing loss^n^	2/24 (8)		7/17 (41)	
		Malignant hyperthermia (MH)				
		History suggestive of MH	4/18 (22)	4/14 (29)	10/18 (56)	3/3 (100)
		No MH^o^	14/18 (78)			
		Unknown^p^	13/31 (42)			
**Disease characteristics**	**Age of presentation**	Features to suggest prenatal onset^q^	12/21 (57)			
		Features present at birth	29/29 (100)			
	**Course of disease**	Static at time of last clinical visit	6/27 (22)			
		Progressive worsening	6/27 (22)			
		Slowly progressive^r^	3/27 (11)			
		Rapidly progressive^s^	3/27 (11)			
		Condition improving	15/27 (56)			
		Unknown	4/31 (13)			

^a^Either unilateral or bilateral ptosis.

^b^One patient had both a high-arched and a cleft palate.

^c^Other abnormalities included chest wall asymmetry, widely spaced nipples, barrel-shaped chest and a Harrison’s sulcus. One patient had a Harrison’s sulcus and chest wall asymmetry.

^d^Respiratory distress that occurred after or beyond the neonatal period.

^e^Atrial septal defect in one patient, and patent ductus arteriosus in one patient (both term gestations).

^f^Two patients had one café-au-lait macule each; the other pigmentary abnormality was not specified.

^g^One patient had thoracic scoliosis documented at birth.

^h^Unilateral or bilateral.

^i^Small and/or large joint hyperlaxity.

^j^Arthrogryposis multiplex congenita as defined by multiple joint contractures affecting two or more areas of the body prior to birth (23).

^k^8/13 (62%) of speech impairment was articulation problems/dysarthria.

^l^Of varying degrees (mild to severe).

^m^Assessed in children age six years or more; all assessed as mild intellectual disability.

^n^One bilateral conductive hearing loss, one hearing loss of unspecified type.

^o^Received general anaesthetic without the development of malignant hyperthermia.

^p^No history of general anaesthesia administered/information not available.

^q^Features to suggest possible prenatal onset included reduced foetal movements, abnormal foetal lie, and polyhydramnios.

^r^One patient had early childhood onset scoliosis which progressed in severity.

^s^One patient died at approximately two years of age from respiratory complications; one patient died at one week of age from respiratory complications; one patient had intraventricular haemorrhage Grade 3 but further history was unknown.

*MH* malignant hyperthermia.

### Previous investigations

#### Brain imaging

Only 10/25 (40%) were reported to have undergone brain imaging (either magnetic resonance imaging or computed tomography). The majority (8/10 (80%)) of these scan results were reported normal; of the two (2/10 (20%)) that were abnormal, one had prominent Sylvian fissures and one showed generalised brain atrophy.

#### Muscle biopsy histology

Muscle biopsies were performed on 16/27 (59%) patients. Histology results were undocumented for 2/16 (12%) patients, and unremarkable for 3/16 (19%). Abnormal muscle biopsy histology results were reported in 11/16 patients (69%). and included documented type 1 muscle fibre predominance (5/11 (45%)), features of non-specific myopathy (3/11 (27%)), mild atrophic fibres (2/11 (18%)), and type 2 muscle fibre predominance (1/11 (9%)).

#### Creatine kinase

Creatine kinase (CK) levels were available for 21/31 (68%) patients. Most patients (16/21 (76%)) had normal CK levels and 4/21 (19%) had raised CK levels, three of whom were sampled in the neonatal period and one at two years of age. Of the four raised CK levels, three were increased two to three times the upper limit of normal for age, and one was increased four to five times the upper limit of normal for age.

#### Genetic testing

Almost all patients (30/31 (97%)) had undergone one or more genetic tests prior to their diagnosis of *STAC3* disorder. Over two thirds (21/31 (68%)) had undergone *SMN1* exon 7 deletion testing for SMA. Table 2 shows the other genetic testing modalities that were performed on the present clinical cohort Patients had between one (10/31 (32%)) and four (5/31 (16%)) additional genetic tests performed prior to the diagnosis of *STAC3* disorder [22]. The majority (21/31 (68%)) of patients had at least one investigation for a chromosome abnormality (fluorescence in situ hybridisation for 22q11.2 deletion syndrome, aneuploidy screen, karyotype and/or chromosome microarray).

**Table 2:** The different genetic tests performed in the diagnostic workup of 31 Southern African patients of African ancestry found to have *STAC3* disorder.

Disorder	Genetic test	Number of patients (%)
Single gene disorders		
Spinal muscular atrophy	*SMN1* genetic analysis (exon 7 deletion)	21 (68)
Autosomal recessive centronuclear myopathy	*RYR1* targeted variant analysis^a^	6 (19)
Prader-Willi syndrome	PWS MS-MLPA	4 (13)
Myotonic dystrophy type 1	Triplet primed PCR	4 (13)
Genetic neuromuscular disorders	Neuromuscular disorder panel^b^	1 (3)
Chromosome abnormalities	Karyotype	11 (35)
	Aneuploidy screen	8 (26)
	Chromosomal microarray	4 (13)
	MLPA^c^	4 (13)
	FISH 22q11.2 deletion	1 (3)

^a^Five common *RYR1* pathogenic variants c.5726_5727delAG (p.Glu1909GlyfsX39), c.6175_6187del13, c.8342_8343delTA (p.Ile2781ArgfsX49), c.11320dupG (p.Ala3774fs), and c.14524 G > A (p.Val4842Met) (22).

^b^Panel did not contain *STAC3*.

^c^Microdeletion syndromes (SALSA MLPA Kit P245) and subtelomeres mix (SALSA MLPA Kit P036) (MRC Holland, Amsterdam, The Netherlands).

*SMN1* Survival of motor neuron 1, *RYR1* Ryanodine receptor 1, *PWS* Prader-Willi syndrome, *MS* methylation specific, *MLPA* multiplex-ligation dependent probe amplification, *FISH* fluorescence in situ hybridisation.

## Discussion

This study describes firstly the detection of *STAC3* disorder in patients of Southern African ancestry from a laboratory cohort who had tested negative for common causes of hypotonia and weakness in infancy and childhood; secondly the carrier frequency estimated in healthy individuals of similar ancestral backgrounds who received WES as part of a study (DDD-Africa); and thirdly the predominantly retrospective file-based analysis of clinical features in patients referred for medical genetic assessment who were found to have *STAC3* disorder.

In laboratory samples, the diagnostic yield of *STAC3* disorder testing was high at 20%, indicating that *STAC3* disorder is a common cause of the “floppy infant” presentation in the local setting. All were homozygous for *STAC3* c.851 G > C (p.Trp284Ser) that is the predominant cause of the condition worldwide.

We estimated the carrier frequency of this allele to be 1 in 56 (95% confidence interval 1:24–1:129), which predicts a birth prevalence of approximately 1 in 12,500 (range 1:2,300–1:65,000), and would result in at least 60 affected babies being born per year in South Africa. By comparison it is estimated that the birth prevalence in Lumbee Native Americans is 1 in 5000 within a total population of 60,000. This suggests that *STAC3* disorder is a more widespread cause of congenital hypotonia than originally reported and may be an important differential diagnosis. Targeted first line *STAC3* testing should be considered particularly in patients of African ancestry.

*STAC3* disorder and its associated common pathogenic variant, were present in patients from Southern Africa of various ethnolinguistic backgrounds. The reason for the relatively high birth prevalence of the condition is unclear. Consanguinity is uncommon in most local populations, and was not present in the families referred for medical genetic assessment. The presence of a common pathogenic variant and, in five patients tested on SNP array, the presence of overlapping regions of homozygosity with a shared haplotype, supports the likelihood of a common ancestral founder mutation, the geographical extent of which remains to be delineated. We identified two cases due to the same homozygous pathogenic variant in individuals from other Southern African countries (Mozambique and Malawi). Gromand et al. [7] described seven cases of *STAC3* disorder due to homozygosity for *STAC3* c.851 G > C in Comoros islanders. The Comoros islands are located in the Mozambique channel, and have a population derived from African and Southeast Asian admixture. The presence in African Americans, with higher frequencies than Europeans, may reflect the African genetic contributions introduced into these populations through the slave trade in the 16th and 17th centuries. The increased carrier frequency observed in the DDD-Africa cohort compared to the allele frequency reported in gnomAD for African/African American populations could be explained by the fact that the African American population has undergone significant genetic admixture. Also, the African population is primarily of West African ancestral origin. The higher frequency observed in Southern Africa may be attributable to the presence of a different ethnolinguistic cohort. This requires further investigation.

To our knowledge this study represents the largest, molecularly homozygous group of patients with *STAC3* disorder with detailed phenotypic frequencies. In keeping with a congenital myopathy, all patients had features suggestive of neuromuscular pathology in the perinatal period: over half (57%) of the patients had features prenatally (such as reduced foetal movements, polyhydramnios or an abnormal foetal lie), and all had suggestive features neonatally. Respiratory distress and feeding difficulties at birth, neonatal hypotonia and neonatal contractures were all noted at high frequencies, suggesting that any combination of these abnormalities should alert attending health care providers to a possible congenital myopathy and, in the African context, *STAC3* disorder. Neonates/infants that survived early respiratory and feeding difficulties tended to show slow clinical improvement.

Growth at birth was variable with just under half born SGA. With age, FTT was common and noted in almost two thirds of the present study group. Patients were typically born with normal lengths, however 17% were born short for gestational age. With time, linear growth continued to be variable but with 45% having short stature of variable onset; a similar frequency (8/18 (44%)) to that reported by Zaharieva et al. [12]. This is most likely a consequence of hypotonia, muscle weakness, and palatal abnormalities, all of which contribute to feeding difficulties and reduced caloric intake. Almost all patients were born with a head circumference within normal ranges; however, some patients were documented to be microcephalic at their last clinical visit, with the age of onset of the microcephaly being unknown in most patients. The timing of the onset of growth failure was very variable.

*STAC3* disorder in the present study was characterised by dysmorphic features in all patients, including a tented upper lip (47%), low-set ears (27%), epicanthic folds (23%), and microretrognathia (20%). Palatal abnormalities were also seen in nearly the entire cohort (93%). Of note, a cleft palate was documented in over one third (39%) of patients. This was a lower frequency than that seen in Gromand et al.’s [7] cohort, in which all seven patients had a cleft palate. Nevertheless, the very high frequency of either high-arched palate (57%) or cleft palate suggests that palatal abnormalities together with features of a congenital myopathy should raise suspicion of *STAC3* disorder.

Systemic abnormalities were common, and most often involved the respiratory, genital, neurological and musculoskeletal systems. Respiratory difficulty that occurred after or extended beyond the neonatal period was noted in almost half (44%) and is likely an underestimate, as respiratory-related data after the neonatal period were available for less than half of the cohort. Almost half (44%) of the male patients with data had either unilateral or bilateral cryptorchidism; this frequency fell between those reported by Gromand et al. [7] and Zaharieva et al. [12], of 20% and 64% respectively.

There was a high frequency (52%) of spine abnormalities in the present study; most frequently scoliosis (30%). Only one patient was noted to have a spinal abnormality at birth (thoracic scoliosis), in contrast to Stamm et al.’s cohort where congenital scoliosis occurred at in 90% [3]. Joint contractures were common, and 59% had TEV deformity/deformities (or a history thereof). This was less frequent than reported by Stamm et al. [3], Gromand et al. [7] and Zaharieva et al. [12], at 77%, 71%, and 67% respectively. Pectus carinatum was documented in a notable 25% of patients. Camptodactyly (40%) and arthrogryposis multiplex congenita (AMC) [23] (38%) were both moderately common. In a hypotonic neonate, joint contractures of any type should raise suspicion of *STAC3* disorder. Joint hyperlaxity was noted in 14% of patients, an interesting and somewhat paradoxical finding as two patients with joint hyperlaxity also had joint contractures. It is possible that the joint hyperlaxity was secondary to hypotonia, but as this was a retrospective study this finding is unclear. Interestingly, Zaharieva et al. (11) also noted joint hyperlaxity in 47% of patients for whom the information was available.

Neurological features that were almost universal included myopathic facies (96%), reduced muscle bulk (86%) and hypotonia (100%). Other common features were globally reduced power (81%), swallowing difficulties (54%), and speech impairment (76%). These are all in keeping with the clinical presentation of congenital myopathies in general. Interestingly, 21% of patients had global deep tendon areflexia, which may lead to clinical confusion with SMA. A further 62% of patients had global hyporeflexia.

Investigations such as brain imaging, muscle biopsy and CK levels all have merit when investigating a ‘floppy baby’; but tend to be non-specific in patients with *STAC3* disorder. In the present cohort, most brain imaging was normal (80%), in contrast to Gromand et al.’s cohort (1/4 (25%)), which identified ventricular dilatation/ventriculomegaly in 3/4 (75%) of patients who had a brain MRI [7]. Muscle biopsy histology was abnormal in most patients who underwent the procedure (69%), with non-specific myopathy features being most common, consistent with previous reports [2]. In a patient with suspected *STAC3* disorder, genetic testing should be performed as the first line investigation, instead of a muscle biopsy which could pose a risk for MH. Creatine kinase levels were only abnormal in four individuals (19%); three of whom were neonates. Previous studies have also shown normal CK levels in patients with NAM [2] and, in general, raised CK levels in the neonatal period are not considered to be a specific indicator of congenital muscular pathology, and can be falsely positive due to intramuscular injections [24]. It was unclear whether the elevated CK level was associated with MH in any of these cases.

Until relatively recently, *STAC3* disorder was not a commonly considered diagnosis in the workup of a patient with congenital myopathy in South Africa. In our experience, patients with *STAC3* disorder have been commonly mistaken as having SMA, *RYR1*-related autosomal recessive centronuclear myopathy, or PWS. This relates in part to awareness of these disorders as a cause of hypotonia in infancy and that genetic testing has long been available for these conditions in the State healthcare sector, whereas *STAC3* disorder became more readily recognised following the introduction of targeted c.851 G > C variant testing in June 2022. The clinical phenotype for *STAC3* disorder has become evident and recognisable, and we recommend that if clinical features of a congenital myopathy are present, together with palatal abnormalities (cleft or high-arched) and joint contracture/s, that genetic testing for *STAC3* disorder should be considered as first line testing, particular in the African context. This may also be the most cost-effective approach to finding a diagnosis in the limited resource settings in southern Africa.

It was noteworthy that a high proportion of cases in the clinical cohort received prior cytogenetic testing. This likely reflects the fact that in addition to hypotonia, infants commonly present with dysmorphism and major congenital anomalies, in particular cleft palate and TEV. A combination of these features implies the presence of multiple congenital anomalies, for which cytogenetic tests such as karyotype and chromosomal microarray are first-line investigations and relatively easily available.

There are numerous reasons why a molecular confirmation of *STAC3* disorder is important. Knowing and understanding the diagnosis assists with optimised medical and surgical patient management, and gives valuable information for the parents/caregivers on long-term sequelae and outcomes. A significant minority of patients (22%), and likely an underestimate, had experienced MH, an important finding as many patients with *STAC3* disorder are likely to undergo surgery for numerous indications such as repairs of cleft palate, club foot, ptosis, or the insertion of feeding tubes. This highlights the importance of a genetic diagnosis to reduce general anaesthesia-related morbidity and mortality. In addition, a confirmed diagnosis allows for accurate genetic counselling on inheritance and recurrence risks, and allows for prenatal genetic testing or preimplantation genetic diagnosis.

*STAC3* disorder is relatively common in individuals with black Southern African ancestry, virtually uniformly due to homozygosity for the common *STAC3* c.851 G > C variant. A high carrier frequency (1 in 56) and high predicted birth prevalence (1 in 12,500), suggests the condition has not been recognised previously as a common African cause of hypotonia and birth defects. A shared haplotype spanning the *STAC3* gene was identified, indicating a potential common origin and founder effect. Similar evidence of founder effects in the black South African population has been documented for various genetic conditions, including Fanconi anaemia, galactosaemia, Bardet Biedl syndrome, MPV17-related mitochondrial neurohepatopathy, Glutaric aciduria type 1 and Hyperphosphatasia with mental retardation syndrome type 4 [25–30]. There are other possible explanations for the fact that *STAC3* disorder is common in this population, even in the presence of a single pathogenic variant and preliminary evidence of a shared haplotype. In particular, heterozygote advantage is a plausible explanation in light of the important physiological role of the STAC3 protein [6].

There is currently limited evidence available to suggest the presence of heterozygote advantage and on balance we think that founder effect (a population bottleneck with genetic drift) is more likely. In this regard, the black Southern African population belong to various Bantu ethnolinguistic groups and represent the most southerly extent of the Bantu expansion, with some Khoe-San admixture [31]. To date, all African countries for which patients with *STAC3* disorder have been described have predominantly Bantu ethnolinguistic populations. Semo et al. [32] found evidence supporting “rapid North-South dispersal of Bantu people along the Indian ocean coast” with evidence of a southward increase in genetic homogeneity with increasing regions of homozygosity. This is consistent with the above-mentioned evidence of founder effect for several genetic disorders [33]. If a founder effect is at play, the exact geographical extent of it requires further exploration.

This group of clinically ascertained patients is the largest yet described and shows similar findings to previous case-series: hypotonia and myopathic facies are universal but most patients have a broader phenotype. Based on our clinical findings, we recommend patients of African ancestry, presenting with features of a congenital myopathy, palatal anomalies and joint contractures (including TEV and AMC), be offered *STAC3* targeted variant genetic testing as a first-line genetic investigation.

The limitations of this study include the cross-sectional nature of the data collection and limited clinical information for some patients, which may lead to underestimation of the frequency of certain clinical features, especially those that are less clinically evident. The laboratory cases may be subject to ascertainment bias as the samples had been previously tested specifically for conditions that present primarily with neonatal hypotonia. However, given that hypotonia is virtually universal in *STAC3* disorder and the limited genetic testing options available at the time, ascertainment may not have been as skewed as expected. Haplotype analysis was not possible on all patients. Prospectively studies on larger cohorts of patients with confirmed *STAC3* disorder are needed to describe the phenotype in more detail, particularly the disease course.

## Supplementary information

Table 3

Table 4

## Data Availability

All data generated or analysed during this study are included in this published article [and its supplementary information files]. The *STAC3* c.851 G > C (p.Trp284Ser) variant described here was submitted to ClinVar on 1 July 2023. This can be viewed under submission Accession SCV004024489, Organization ID 508172 (https://www.ncbi.nlm.nih.gov/clinvar/RCV000074400/).
