# Supplementary material for: STAC3 disorder: a common cause of congenital hypotonia in Southern African patients
Source: Eur J Hum Genet. 2024 Jun 1;33(1):14–23. doi: 10.1038/s41431-024-01644-5 (PMC11711498; doi:10.1038/s41431-024-01644-5)
Supplement: Supplementary file 1 — Table 3 [file 41431_2024_1644_MOESM1_ESM.docx]

**Supplementary data**

**Table 3.** Frequency of perinatal characteristics of Southern African patients of African ancestry with *STAC3* disorder due to apparent homozygosity for a pathogenic variant (c.851G>C, p.Trp284Ser) in *STAC3*

| **Perinatal characteristic^1^** | | | **Number (%)** |
| --- | --- | --- | --- |
| **Fetal movements** | Normal | | 13/21 (62) |
|  | Reduced | | 8/21 (38) |
| **Fetal lie** | Normal | | 18/23 (78) |
|  | Abnormal | | 5/23 (22) |
| **Liquor volume** | No polyhydramnios | | 17/18 (94) |
|  | Polyhydramnios | | 1/18 (6) |
| **Progress of labour** | Normal | | 9/19 (47) |
|  | Poor progress | | 4/19 (21) |
|  | Not applicable | | 6/19 (32) |
| **Fetal distress** | No | | 15/25 (60) |
|  | Yes | | 10/25 (40) |
| **Gestational age at birth** | Premature | | 4/29 (14) |
|  | Term | | 25/29 (86) |
| **Assisted birth** | No | | 23/25 (92) |
|  | Yes | | 2/25 (8) |
| **Apgar scores** | 1 minute | 0 -3 | 1/21 (5) |
|  |  | 4 - 6 | 12/21 (57) |
|  |  | 7-10 | 8/21 (38) |
|  | 5 minute | 0 -3 | 0/20 (0) |
|  |  | 4 - 6 | 5/20 (25) |
|  |  | 7-10 | 15/20 (75) |
|  | 10 minute | 0 -3 | 0/4 (0) |
|  |  | 4 - 6 | 1/4 (25) |
|  |  | 7-10 | 3/4 (75) |
| **Neonatal respiratory distress** | No | | 8/26 (31) |
|  | Yes | | 18/26 (69) |
| **Neonatal feeding difficulties** | No | | 5/28 (18) |
|  | Yes | | 23/28 (82) |
| **Neonatal hypotonia** | No | | 1/28 (4) |
|  | Yes | | 27/28 (96) |
| **Weak cry at birth** | No | | 1/17 (6) |
|  | Yes | | 16/17 (94) |
| **Neonatal contractures** | No | | 9/29 (31) |
|  | Yes | | 20/29 (69) |
| **Birth growth parameters** | Weight | Appropriate for gestational age | 14/25 (56) |
|  |  | Small for gestational age | 11/25 (44) |
|  |  | Large for gestational age | 0/25 (0) |
|  | Length | Normal | 13/18 (72) |
|  |  | Short stature^2^ | 3/18 (17) |
|  |  | Tall stature | 2/18 (11) |
|  | Head circumference | Normal | 18/19 (95) |
|  |  | Microcephaly | 0/19 (0) |
|  |  | Macrocephaly^3^ | 1/19 (5) |

**Notes**

^1^ For which data were available

^2^ Two patients had length between -2.00 and -3.00 standard deviation (SD) scores and one patient had a length greater than 3.00 SD scores below the mean

^3^ Macrocephaly great than the 97^th^ centile and greater than three SD scores above the mean.
